# Thermodynamic parameters of U (VI) sorption onto soils in aquatic systems

**DOI:** 10.1186/2193-1801-2-530

**Published:** 2013-10-17

**Authors:** Ajay Kumar, Sabyasachi Rout, Malay Ghosh, Rakesh Kumar Singhal, Pazhayath Mana Ravi

**Affiliations:** Health Physics Division, Bhabha Atomic Research Centre, Mumbai, India; Health Physics Unit, Bhabha Atomic Research Centre, Mysore, India; Analytical Chemistry Division, Bhabha Atomic Research Centre, Mumbai, India

**Keywords:** Soil, Groundwater, Deionised water, Distribution coefficients, Uranium

## Abstract

The thermodynamic parameters viz. the standard free energy (∆Gº), Standard enthalpy change (∆Hº) and standard entropy change (∆Sº) were determined using the obtained values of distribution coefficient (k_d_) of U (VI) in two different types of soils (agricultural and undisturbed) by conducting a batch equilibrium experiment with aqueous media (groundwater and deionised water) at two different temperatures 25°C and 50°C. The obtained distribution coefficients (k_d_) values of U for undisturbed soil in groundwater showed about 75% higher than in agricultural soil at 25°C while in deionised water, these values were highly insignificant for both soils indicating that groundwater was observed to be more favorable for high surface sorption. At 50°C, the increased k_d_ values in both soils revealed that solubility of U decreased with increasing temperature. Batch adsorption results indicated that U sorption onto soils was promoted at higher temperature and an endothermic and spontaneous interfacial process. The high positive values of ∆Sº for agricultural soil suggested a decrease in sorption capacity of U in that soil due to increased randomness at solid-solution interface. The low sorption onto agricultural soil may be due to presence of high amount of coarse particles in the form of sand (56%). Geochemical modeling predicted that mixed hydroxo-carbonato complexes of uranium were the most stable and abundant complexes in equilibrium solution during experimental.

## Introduction

The sorption of uranium onto soil is a result of several processes such as adsorption, chemisorptions and ion exchange (Sheppard et al. 1987). The behavior and mobility of radionuclides in soil is a major consideration for distribution coefficients and is influenced by many variables. The distribution coefficient characteristics of radionuclides have been observed to vary depending on soil properties such as texture, organic matter content, bacterial action, pH, redox potential and physicochemical speciation. Because of its dependence on many soil properties, the value of the distribution coefficients for a specific radionuclide can range over several orders of magnitude under different conditions.

Soils contain a number of radionuclide adsorbing components in the silt and clay fractions. The most important for the sorption of radionuclides are minerals such as smectite, illite, vermiculite, chlorite, allophone and imogolite as well as the oxides and hydroxides of silica, aluminium, iron and manganese. The adsorption is due to the charge at the surface of these soil constituents and the three-dimensional structure of the adsorbing mineral. Clay minerals carry different kinds of charge, a “variable charge” which can be either negative or positive and a “permanent charge” which is merely negative. A permanent negative charge results from a replacement of cations by cations with a lower positive charge within the mineral lattice. This process is independent of the pH value and results in a general ability to adsorb cations.

Uranium as hexavalent state is the thermodynamically stable in oxic groundwater and interacts strongly with solid phases. The transport of uranium in the groundwater is governed by its interactions with adsorbed phases and a variety of sorption reactions are involved in the dynamic of uranium in soil. The increase in U(VI) adsorption onto soil from acidic to near neutral pH values is a consequence of the dominant U(VI) aqueous species being cationic and neutral over this pH range. However, the subsequent decrease in U(VI) adsorption with increasing basic pH values results from the dominant U(VI) aqueous species being anionic U(VI) carbonate complexes (Tripathi 1984, Hsi and Langmuir, 1985, Waite et al.^1994^, McKinley et al. 1995, Turner et al. 1996). In the absence of dissolved carbonate, uranium sorption to iron oxide and clay minerals has been shown to be extensive and remain at a maximum at pH values near and above neutral pH (Hsi and Langmuir 1985). However, in the presence of carbonate and organic complexants, U(VI) adsorption has been shown to be substantially reduced or inhibited. Even differences in partial pressures of CO_2_ have a major effect on uranium adsorption at neutral pH conditions. Waite et al. (1994), showed that the percent of U(VI) adsorbed onto ferrihydrite decreases from 97 to 38% when CO_2_ is increased from ambient (0.03%) to elevated (1%) partial pressures. Kaplan and Serne (1995) noted that U (VI) adsorption typically increases with increasing ionic strength of an oxidized aqueous solution. The presence of increasing concentrations of other dissolved ions, such as Ca^2+^, Mg^2+^ and K^+^ will displace the U (VI) ions adsorbed onto mineral surface sites and release U(VI) into the aqueous solution. Therefore, the mobility of U(VI) is expected to increase in high ionic-strength solutions.

Naturally occurring organic matter in soils is also important in the adsorption of uranium. Several mechanisms have been proposed for U(VI) adsorption by organic matter (Kaplan and Serne 1995). The adsorption of uranium to humic substances may occur through ion exchange and complexation processes that result in the formation of stable U (VI) complexes involving the acidic functional groups (Idiz et al. 1986, Shanbhag and Choppin 1981). Alternatively, Nash et al. (1981) has suggested that organic material may act to reduce dissolved U (VI) species to U(IV). Organic matter generally reduces anion adsorption due to the formation of organic coatings on the surface anion adsorbing minerals.

Distribution coefficient is a useful parameter for comparing the sorptive capacity of different soils or materials for any particular ion, when they are measured under the same experimental conditions. The mobility of metals in the environment are directly related to their partitioning between solid and liquid phases and therefore, are directly related to their distribution coefficients, which indicate the capability of a sorbent to retain a solute and the extent of its movement to the liquid. Since data of thermodynamic parameters for U sorption in Indian soils are limited. Therefore, the objective of the present study is to obtain the distribution coefficients of U in soils (agricultural and undisturbed) under different aqueous media (groundwater and deionised water)) at two particular temperatures using a batch equilibrium experiment and subsequently to determine the thermodynamic parameters viz. ∆Gº, ∆Hº and ∆Sº.

### Experimental

Two bulk composite surface (depth upto15cm) soil samples representing undisturbed and agricultural soil of 1 kg each were collected from two different sites in Mumbai (India). The collected soil samples were dried at 110°C for 24 h, powdered, homogenized and sieved through 110 mesh sizes. The powdered samples were thoroughly mixed with each other and washed thrice with deionised water for 7 days. The solid phase was allowed to settle by gravity and the washing solution was discarded. After washing, samples were further dried at 110°C, placed in conical flasks and stored as stock samples for experimental work.

A batch equilibrium experiment was conducted to determine the distribution coefficient of U in both soils under two different aqueous media viz. groundwater and deionised water. The distribution coefficient was calculated using batch method formula. Each of 5 g dried soil samples was placed in PTFE containers with lid to avoid significant sorption at higher pH on glass ware and equilibrated for 7 days with each of 150 mL groundwater and deionised water containing 10 mgL^-1^ of uranium prepared from standard solutions (1 gL^-1^) of uranyl nitrate hexahydrate (USA make) followed by shaking using shaker at 25°± 1°C and 50°± 2°C in an incubator. After equilibration time, the samples of each set were centrifuged, filtered through 0.45 μm filter paper and concentration of U in the equilibrium solution was determined using laser fluorimetery (Quantalase Indore, India) in which a pulsed nitrogen laser is used to excite uranyl species present in the samples at 337.1 nm which on de-excitation gives out fluorescence peaking at 496, 516 and 540 nm. Finally standard addition technique was followed for the estimation of uranium in the samples. The instrument was calibrated in the range of 1–100 μg/L. 5% sodium pyrophosphate in ultra pure water was used as fluorescence reagent. Relative standard deviation (RSD) was calculated to be 3–8%. Quality assurance was carried out by spike recovery, replicate analysis and cross method checking. The pH of the equilibrated solution ranged from 7.8 to 8.0. Sample preparations, procedures and conditions for the experiments were the same to achieve reproducible results in order to make comparisons. The ionic composition of equilibrium solutions of both aqueous media was determined by Ion-chromatography system (DIONEX, 600) using conductivity suppressor as given in Table 1. HCO_3_^-^ and CO_3_^2-^ were estimated titrimetrically using autotitrator (Metrohm-798 MPT Titrino). Using an Eh -pH equation for water electrode, the Eh value determined to be +0.8 V at P_O2_ = 1 atm. The particle size distribution of soil samples was determined using a laser diffraction particle size analyzer (CILAS, France, Model 1190). Prior to the experiments, U concentrations in groundwater, deionised water and soils as a background concentration were also determined. The activity levels of uranium (^238^U) in soils before experiment were estimated using Gamma spectrometry system (HPGe, 50% relative efficiency) after attaining the secular equilibrium between ^226^Ra, ^214^Pb and ^214^Bi in the ^238^U decay chain. All the experimental data were the averages of duplicate or triplicate measurements.

**Table 1:** **Average value of ionic composition in equilibrium solutions of soils in two different aqueous media at 25°C**

Ionic components	Values	
	Groundwater	Deionised water
U (μg L^-1^ )	78	111
Cl^-1^ (mgL^-1^)	12	6
NO_3_^-1^(mgL^-1^)	2	ND
SO_4_^-2^(mgL^-1^)	8	2
HCO_3_^-1^ (mgL^-1^)	308	ND
CO_3_^2-^(mg/L)	1.6	ND
Na^+1^(mgL^-1^)	14	5
K^+1^(mgL^-1^)	1.8	1.2
Mg^+2^(mgL^-1^)	6	2.4
Ca^+2^(mgL^-1^)	7	2

FTIR **(Bruker, Germany)** spectra for both soils were obtained using platinum attenuated total reflection **(ATR)** technique. All spectra were recorded using a resolution of 4 cm^-1^ (wave number) and equal measurement conditions (3900–450 cm^-1^, 40 scans, scans means 16 repetitions of a single FT-IR measurement).

The total carbon and nitrogen in soils were estimated using C H N S-O elemental analyser (Flash EA 1112 Series, Thermo Finnigan, Italy).

The sorption of uranium onto soil expressed in terms of distribution coefficient (k_d_) was calculated according to Equation (1) **[**Ajay Kumar et al. 2013, Lu et al. 2011, Chen et al. 2008, Hu et al. 2011, Zuo et al. 2011)1

where, *C*_*i*,_ = initial concentration of U in the solution; *C*_*e*_ = concentration of U in the solution after reaching equilibrium, *V* = volume of the contact solution (mL) and *m* = mass of the soil (g).

Equilibrium distribution of aqueous species of U at major ionic components of equilibrium solution was calculated by the geochemical model which is based on an extensive thermodynamic data base of uranium complexes with various ligands such as hydroxide, chloride, nitrate, carbonate, fluoride, sulphate, bicarbonate etc.

The thermodynamic parameters can be determined from the thermodynamic equilibrium constant, K^0^ (or the thermodynamic distribution coefficient) which is defined as2

where *a*_*s*_ = activity of adsorbed U on soil, *a*_*e*_ = activity of U in solution at equilibrium, *γ*_*s*_ = the activity coefficient of adsorbed U, *γ*_*e*_ = the activity coefficient of U in equilibrium solution, *C*_*s*_ = concentration of adsorbed U on soil.

The expression of K^0^ can be simplified by assuming that the concentration in the solution approaches zero resulting in *C*_*s*_→0 and *C*_*e*_→0 and the activity coefficients approach unity at these very low concentrations (Biggar and Cheung 1973, Calvet R. 1989). Equation (2) can be written as:3

Hence, ∆G^0^ (kJ mol^-1^) at temperature T (in Kelvin) was calculated as follows:4

where *R* is the gas constant (8.314 Jmol^-1^K^-1^), T is the temperature in Kelvin.

Moreover, since the adsorption isotherms have been measured at two temperatures, the heat of adsorption can be calculated. The temperature dependency of distribution coefficient (*k*_*d*_) obeyed the van’t Hoff equation which can be written in the form of ∆H^0^5

and *k*_*d(1)*_ and *k*_*d(2)*_ are the distribution coefficients at two temperatures *T*_*1*_ and *T*_*2*_ (in Kelvin) respectively.

∆S^0^ (J mol^-1^ K^-1^) was calculated as (Hu et al. 2010, Yang et al. 2010, 2012)6

## Results and discussions

### Soil texture

Agricultural soils collected from the depth of <15 cm at the sampling site were sandy silt loam in the form of 3% clay (< 2 μm), 41% silt (< 2 - > 63 μm) and 56% sand (> 63 μm) whereas undisturbed soils were found to be silty-sand loam with the distribution as 5% clay, 61% silt and 34% sand. The mean of bulk density, porosity and moisture content of both soils were determined to be 1.64 gm/cc, 36% and 2.21% respectively. Average soil particle density was assumed to be 2640 kg/m^3^.

### Uranium sorption onto soils at two different temperatures

In the previous study, sorption of U in seawater-sediment system was studied using batch method and concluded that distribution coefficient (k_d_) are not only dependent on sediment properties but also on the kinds of minerals in sediments (Ajay Kumar et al. 2013). In this study, the sorption of U onto undisturbed and agricultural soil in terms of k_d_ values was initially examined in groundwater and deionised water system at two different temperatures under the similar laboratory conditions. Table 2 illustrates the physical properties of soils including k_d_ values of U in two different aqueous media at 25°C and 50°C.

**Table 2:** **Soil characteristics including k**_**d**_ **values of U under two different aqueous media at 25°C and 50°C**

Soil samples	Bulk density (gm/cc)	Porosity (%)	Moisture content (%)	Sand (%)	Silt (%)	Clay (%)	N (%)	C (%)	Distribution coefficients (k_d_, Lkg^-1^)			
									Deionised water		Groundwater	
									25°C	50°C	25°C	50°C
Undisturbed soil	1.72	35	1.62	34	61	5	2.10	1.90	2725± 342	3543± 365	5246 ± 460	5612 ± 572
Agricultural soil	1.67	37	2.80	56	41	3	1.74	0.84	2620± 235	4362± 325	3008±324	4982±452

In undisturbed soil, at 25°C, the average extraction rate of U in groundwater and deionised water was determined to be 0.054% per day and 0.11% per day respectively whereas for agricultural soil, there was an almost similar extraction rate of 0.10% per day in both aqueous medium. Similarly, the average extraction rate at 50°C, for undisturbed soil was found to be 0.051% per day and 0.08% per day for groundwater and deionised water respectively and for agricultural soil, the extraction rate was about 0.06% per day in both medium. At 25°C, for undisturbed soil, the extraction rate in groundwater was about 50% slower than in deionised water suggesting that groundwater was observed to be more favorable for high surface sorption of U onto such soils. Prior to the experiments, mean concentrations of U in groundwater, deionised water and soils as a background concentration were determined to be 3 μgL^-1^, < 0.2 μgL^-1^ and 1.7 μgg^-1^respectively.

The ratio of obtained k_d_ values of U for undisturbed soil in groundwater to deionised water was found to be 1.92 and 1.58 at 25°C and 50°C respectively whereas for agricultural soil this ratio was obtained to be almost constant as 1.14 for both temperature. This clearly shows that the sorption of uranium onto undisturbed soil was found to be stronger than agricultural soil in both aqueous media at 25°C. The high sorption onto undisturbed soil may be due to presence of high amount of finer particles in the form of silt (61%) and clay (5%) and less content of sand (34%). The finer the particles, the greater the exchange surfaces of U and higher the binding capacity. The low sorption onto agricultural soil may be due to presence of high amount of coarse particles in the form of sand (56%). Due to high content of sand, availability of exchange surfaces for U in soil is small and subsequently it released into the groundwater leading to low k_d_. The another reason for low k_d_ in agricultural soil might be due to low abundances of total carbon content caused for the poor sorption and complexation processes on organic soil constituents. The ratio of carbon content in undisturbed to agricultural soil was observed to be 2.26.

At high temperature (50°C), enhanced k_d_ values in both soils indicates that sorption increases as temperature increases. This may be due to increase in diffusion rate of U(VI) into the pores of soils (Chen et al. 2009; Zhao et al. 2010). Changes in the soils pore sizes as well as an increase in the number of active sorption sites due to breaking of some internal bonds near soil surface edge are generally expected at higher temperatures. Therefore, the increase in temperature may result in the increase in proportion and concentration of U(VI) in solution, the affinity of U(VI) to the soil surface and the potential charge of soil surface (Ghosh et al. 2008).

Kaplan et al. (1998) investigated the adsorption of U (VI) on natural sediment (a silty loam and very coarse sand) containing carbonate minerals in groundwater system and found the k_d_ values greater than 400 Lkg^-1^ at pH > 10. Gamerdinger et al. (1998) conducted a series of experiments to measure the adsorption of U(VI) on sediments (medium coarse sand, fine sand and silt loam) in groundwater at pH 8.4 under partial moisture saturation conditions and found as increasing trend of k_d_ values with moisture saturation content. US EPA 1999, has reported the soil- water k_d_ values in the range of 0.4 – 250000 Lkg^-1^ at pH 8 for U in the look-up-table.

### Thermodynamic studies

The determination of the thermodynamic parameters (∆Hº, ∆Sº and ∆Gº) for U(VI) as given in Table 3 can provide mechanism insights into U(VI) adsorption onto soils. The values of the standard enthalpy change (∆Hº) were positive in both soils, indicating that it is an endothermic process for U adsorption onto soils. One possible interpretation for the endothermic process is that U(VI) ions are well solvated in water. In order to be adsorbed onto soils, U (VI) ions are denuded of their hydration sheath to some extent and this dehydration process needs energy. It is assumed that the needed energy for dehydration exceeds the exothermicity of the ions attaching to soil surfaces. The implicit assumption herein is that the adsorbed U(VI) ions are less hydrated than those in solution. The removal of water molecules from U(VI) ions is essentially an endothermic process and the endothermicity of the desolvation process exceeds the enthalpy of sorption to a considerable extent (Yang et al. 2010, 2011a, Hu et al. 2010).

**Table 3:** **The Obtained thermodynamic parameters for U sorption onto soils in two different aqueous media at 25°C and 50°C**

Soil samples	∆G^0^(kJ/mol)				∆H^0^(kJ/mol)		∆S^0^(J/ mol/°K)			
	Deionised water		Groundwater		Deionised water	Groundwater	Deionised water		Groundwater	
	25°C	50°C	25°C	50°C			25°C	50°C	25°C	50°C
Undisturbed soil	-19.60	-20.45	-21.22	-21.39	8.41	2.16	94	89	78	73
Agricultural soil	-19.50	-20.76	-19.84	-21.09	16.31	16.15	120	115	121	115

The values of the Gibbs free energy change (∆Gº) were all negative at two temperatures studied herein as expected for a spontaneous process under our experimental conditions. The higher the reaction temperature, the more negative the value of ∆Gº, indicating that the adsorption reaction is more favorable at elevated temperatures (Hu et al. 2010). At higher temperature, U(VI) ions are readily dehydrated and thereby their sorption becomes more favorable. The ∆Gº values were observed to be relatively higher for undisturbed soils at both temperatures which might be due to high silt and clay content and low moisture content.

However, the values of the standard entropy change (∆Sº) in soils were all positive for U(VI) sorption onto soils, which indicates that during the whole adsorption process, some structural changes occurs on soils surface and thus leading to an increase in the disorderness at the soil- water interface (Hu et al. 2010). In addition, whether or not a surface adsorption reaction is an associative or dissociative mechanism, strongly depends on the value of ∆Sº. When the value of ∆Sº is higher than -10 kJ mol^-1^, a dissociative mechanism controls adsorption (Hu et al. 2010, Yang et al. 2010, 2012). The large ∆Sº values at the two temperatures herein suggests that a dissociative mechanism is responsible for U(VI) adsorption onto soils.

The higher values obtained for ∆Sº in agricultural soil than in undisturbed soil confirmed that agricultural soil has comparatively low sorption capacity for U which leads to less k_d_. Furthermore, the decreased ∆Sº values at elevated temperature in both soils revealed the more efficient sorption at higher temperature (Yang et al. 2009, 2011b, 2011c, Zhao et al. 2010).

### FTIR spectroscopy

**Figure 1:**
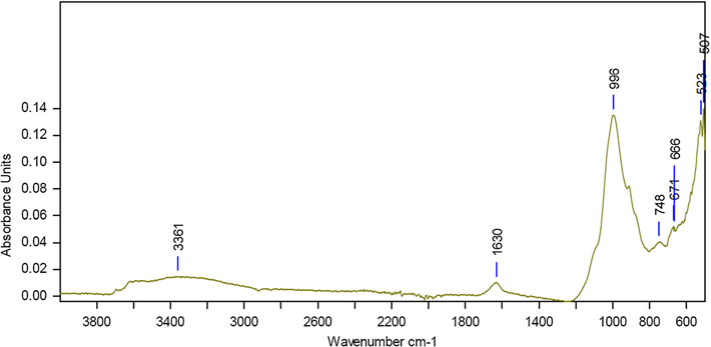
**FTIR spectra of undisturbed soil at 25°C.**

**Figure 2:**
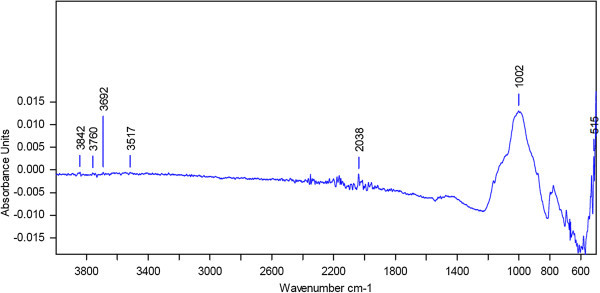
**FTIR spectra of agricultural soil at 25°C.**

The intensity of absorption bands depends on the amount of absorbing functional groups. It reveals that a high absorption indicates a high content of the corresponding functional group whereas a low absorption band indicates a low content of this group. The FTIR spectra of undisturbed and agricultural soil are depicted in Figures 1 and 2 respectively. The wave number indicates the kind of functional groups which are due to absorption. The FTIR spectrum of undisturbed soil revealed the main absorption bands at 3361 cm^-1^ that represents H-bonded OH groups (alcohols, phenols, water molecules), the band at 1630 cm^-1^ related to C=O stretching vibration of carboxylic and ketonic groups and the band at 996 cm^-1^due to symmetric stretching vibration of silicate group, However the agricultural soil did not show any significant intensity of absorption band except at 1002 cm^-1^ which also indicates a silicate group.

### Speciation of U in equilibrium solution

**Figure 3:**
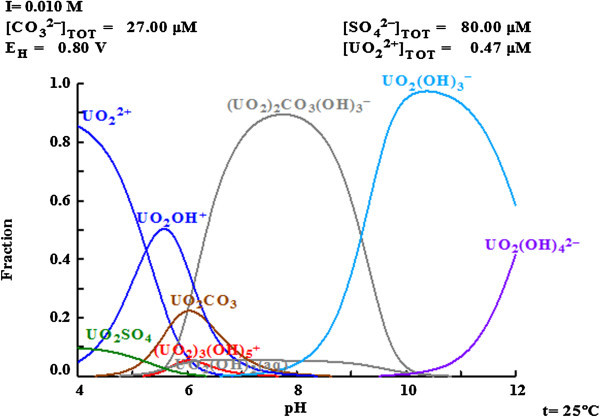
**The fraction of uranium species as a function of pH in equilibrium solution of ground water.**

The mean concentration of uranium in equilibrium solution of both soils in deionised water at 25°C was determined to be 111 μgL^-1^(~ 4.7 × 10^-7^ M) whereas for groundwater, the mean value was 78 μgL^-1^(~ 3.3 × 10^-7^ M). Figure 3 shows the equilibrium aqueous speciation of U as a function of pH in groundwater. The predicted aqueous species as (UO_2_)_2_CO_3_ (OH)_3_^-^(aq) accounted for about 90% of the total dissolved U at measured pH range. Since the major aqueous species is mixed hydroxo-carbonato complexes of U, therefore it is assumed that this complex is sorbed onto soil surface and caused the removal of U. Under alkaline conditions, uranium forms anionic complexes and therefore cannot be efficiently sorbed onto soil surface.

## Conclusions

The results derived from this work indicate that the thermodynamic parameters are related to both the nature of sorbate and the nature of solid particles. The thermodynamic analysis of U(VI) adsorption indicates that the surface reaction of U(VI) adsorption onto soils is an endothermic and spontaneous process. Results of thermodynamic studies revealed that U sorption reaction in both soils were less susceptible to U toxicity due to obtaining high k_d_ values at high temperature (50°C). But at ambient temperature (25°C), agricultural soils were more prone to U toxicity than undisturbed soil indicating that such soils will pose more problems of U contamination and its toxicity to the plants. Thus soil properties, nature of pollutant and soil environment particularly temperature needs to be considered in the assessment of soil quality. The higher positive values obtained for ∆Sº in agricultural soil also confirmed that agricultural soil has less sorption capacity due to high degree of randomness at solid-solution interface during the sorption of U. The data and modeling calculations illustrate that it is important to take into account the effect of geochemical parameters on U aqueous speciation when predicting its sorption and mobility at contaminated soil. The findings in this study are quite important to understand the physicochemical behavior of the interested radionuclides in the natural environment.
